# Chemotherapeutic Drugs Induce Different Gut Microbiota Disorder Pattern and NOD/RIP2/NF-κB Signaling Pathway Activation That Lead to Different Degrees of Intestinal Injury

**DOI:** 10.1128/spectrum.01677-22

**Published:** 2022-10-12

**Authors:** Bin Huang, Mengxuan Gui, Zhuona Ni, Yanbin He, Jinyan Zhao, Jun Peng, Jiumao Lin

**Affiliations:** a Academy of Integrative Medicine of Fujian University of Traditional Chinese Medicine, Fuzhou, Fujian, People’s Republic of China; b Fujian Key Laboratory of Integrative Medicine on Geriatrics, Fujian University of Traditional Chinese Medicine, Fuzhou, Fujian, People’s Republic of China; c Key Laboratory of Integrative Medicine of Fujian Province University, Fujian University of Traditional Chinese Medicine, Fuzhou, Fujian, People’s Republic of China; University of Prince Edward Island

**Keywords:** colorectal cancer, chemotherapeutic drugs, intestinal injury, 5-fluorouracil, irinotecan, oxaliplatin, calcium folinate, gut microbiota disorder, NOD/RIP2/NF-κB signaling pathway

## Abstract

5-Fluorouracil (5-FU), irinotecan (CPT-11), oxaliplatin (L-OHP), and calcium folinate (CF) are widely used chemotherapeutic drugs to treat colorectal cancer. However, chemotherapeutic use is often accompanied by intestinal inflammation and gut microbiota disorder. Changes in gut microbiota may destroy the intestinal barrier, which contributes to the severity of intestinal injury. However, intestinal injury and gut microbiota disorder have yet to be compared among 5-FU, CPT-11, L-OHP, and CF in detail, thereby limiting the development of targeted detoxification therapy after chemotherapy. In this study, a model of chemotherapy-induced intestinal injury in tumor-bearing mice was established by intraperitoneally injecting chemotherapeutic drugs at a clinically equivalent dose. 16S rRNA gene sequencing was used to detect gut microbiota. We found that 5-FU, CPT-11, and l-OHP caused intestinal injury, inflammatory cytokine (gamma interferon [IFN-γ], tumor necrosis factor alpha [TNF-α], interleukin-1β [IL-1β], and IL-6) secretion, and gut microbiota disorder. We established a complex but clear network between the pattern of changes in gut microbiota and degree of intestinal damage induced by different chemotherapeutic drugs. L-OHP caused the most severe damage in the intestine and disorder of the gut microbiota and showed a considerable overlap of the pattern of changes in microbiota with 5-FU and CPT-11. Analysis by Phylogenetic Investigation of Communities by Reconstruction of Unobserved States (PICRUSt v.1.0) showed that the microbiota disorder pattern induced by 5-FU, CPT-11, and L-OHP was related to the NOD-like signaling pathway. Therefore, we detected the protein expression of the NOD/RIP2/NF-κB signaling pathway and found that L-OHP most activated this pathway. Redundancy analysis/canonical correlation analysis (RDA/CCA) revealed that *Bifidobacterium*, *Akkermansia*, *Allobaculum*, *Catenibacterium*, *Mucispirillum*, *Turicibacter*, *Helicobacter*, Proteus, Escherichia *Shigella*, *Alloprevotealla*, *Vagococcus*, Streptococcus, and “*Candidatus* Saccharimonas” were highly correlated with the NOD/RIP2/NF-κB signaling pathway and influenced by chemotherapeutic drugs.

**IMPORTANCE** Chemotherapy-induced intestinal injury limits the clinical use of drugs. Intestinal injury involves multiple signaling pathways and gut microbiota disruption. Our results suggested that the degree of intestinal injury caused by different drugs of the first-line colorectal chemotherapy regimen is related to the pattern of changes in microbiota. The activation of the NOD/RIP2/NF-κB signaling pathway was also related to the pattern of changes in microbiota. l-OHP caused the most severe damage to the intestine and showed a considerable overlap of the pattern of changes in microbiota with 5-FU and CPT-11. Thirteen bacterial genera were related to different levels of intestinal injury and correlated with the NOD/RIP2/NF-κB pathway. Here, we established a network of different chemotherapeutic drugs, gut microbiota, and the NOD/RIP2/NF-κB signaling pathway. This study likely provided a new basis for further elucidating the mechanism and clinical treatment of intestinal injury caused by chemotherapy.

## INTRODUCTION

Cancer is a major public health problem. Colorectal cancer has the third-highest incidence and the fifth-highest mortality rate among cancers (1). Few patients with colorectal cancer are diagnosed at clinical stage I, but most are diagnosed in advanced stages (2, 3). Eliminating colorectal cancer via surgical resection is difficult because of the high probability of tumor recurrence and metastasis. Because of the development of neoadjuvant and adjuvant chemotherapy, patients can take appropriate chemotherapeutic drugs for adjuvant treatment before or after surgery to improve the surgical resection rate and prevent tumor recurrence or metastasis (3). 5-Fluorouracil (5-FU), irinotecan (CPT-11), oxaliplatin (L-OHP), and calcium folinate (CF) are the most widely used chemotherapeutic drugs to treat colorectal cancer (4). 5-FU, CPT-11, and L-OHP inhibit tumor cell proliferation and induce apoptosis by interfering with DNA and RNA synthesis in tumor cells (5). CF has no antitumor activity, but it is commonly used in the FOLFOX (folinic acid, 5-FU, and L-OHP) regimen to increase the efficacy of 5-FU (6).

The interference of chemotherapy in DNA and RNA synthesis targets not only tumor cells but also normal cells, consequently leading to a series of side effects. Approximately 50% to 80% of patients with colorectal cancer treated with 5-FU, CPT-11, or L-OHP develop chemotherapy-induced intestinal injury with severe diarrhea, nausea, vomiting, anorexia, and weight loss (7). The debilitating effects of intestinal injury include pain, increased length of hospitalization, decreased quality of patients’ life, modification of antineoplastic treatment regimens, increased risk of systemic infections, and even death (8–10).

Chemotherapy-induced intestinal injury has several contributing pathogenic elements, including crypt epithelium apoptosis, hypoproliferation, abnormal inflammation (11, 12), and gut microbiota disorder (13, 14). Changes in the gut microbiota may destroy the intestinal barrier and cause serious intestinal injury (15). Nucleotide binding oligomerization domain containing 1 (NOD1) and NOD2 can be activated by exogenous microbiota; the NOD/receptor-interacting protein (RIP2)/NF-κB signaling pathway plays an essential role in inflammation (16). After the activation of this pathway, the secretion of several inflammatory cytokines, including tumor necrosis factor alpha (TNF-α), interleukin-1β (IL-1β), IL-6, and interferon gamma (IFN-γ), is upregulated (17). Therefore, this study aimed to investigate whether chemotherapeutic drugs (5-FU, CPT-11, L-OHP, and CF) would lead to different pathophysiological properties of intestinal injury, patterns of gut microbiota disorder, and molecular mechanisms. Understanding these changes could provide new ideas and directions for the targeted treatment of intestinal injury induced by colorectal cancer chemotherapy.

## RESULTS

### Influence of different chemotherapeutic drugs on the mortality, body weight loss, diarrhea index, and fecal occult blood score of mice.

**FIG 1:**
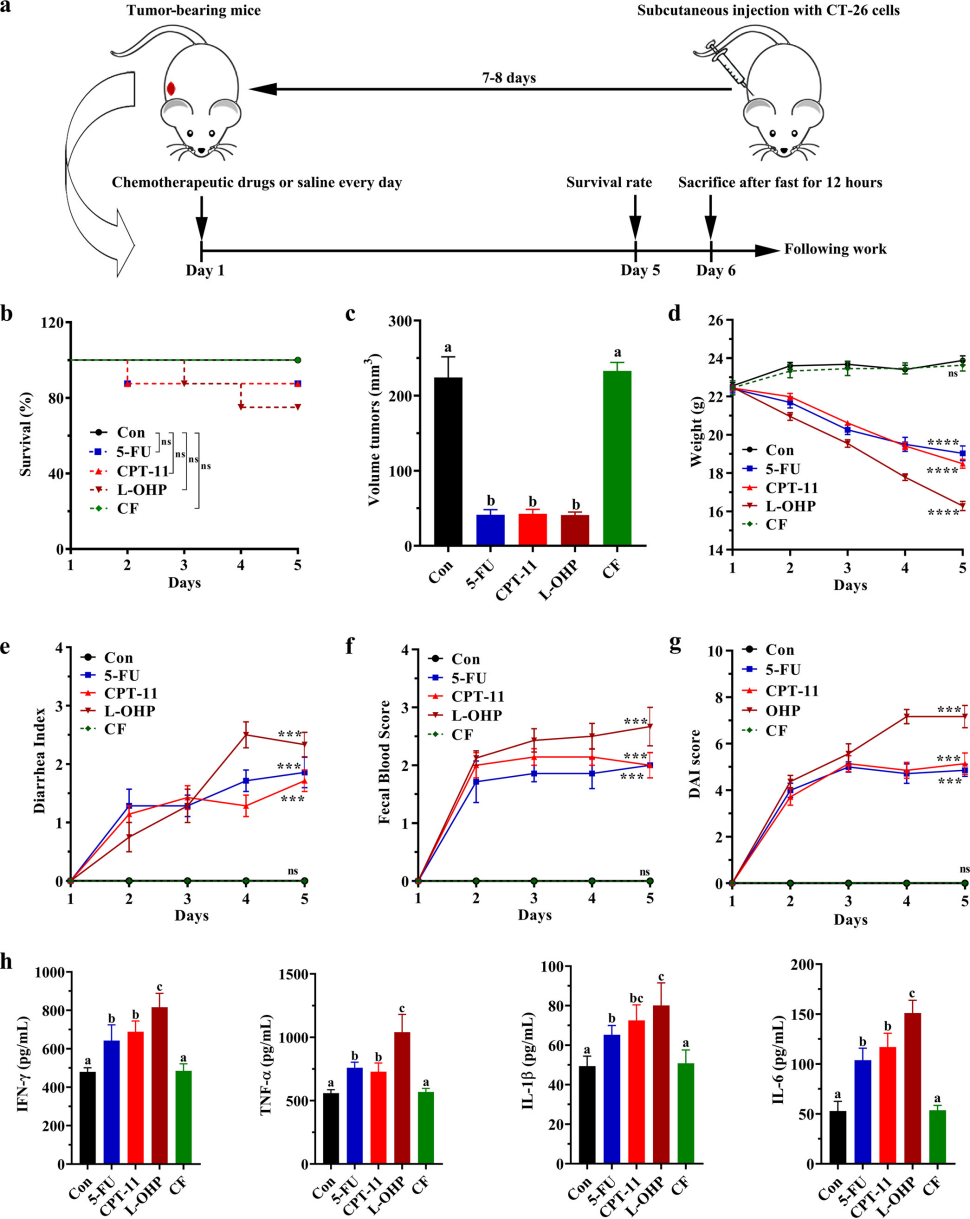
Different chemotherapeutic drugs induced intestinal mucositis in mice. (a) Experimental procedure for intestinal mucositis model administration; (b to d) mortality (b), tumor volume (c), and body weight (d) of the mice in different groups; (e to g) average diarrhea index (e), fecal blood score (f), and DAI (g) of the mice; (h) expression of inflammatory cytokines (IFN-γ, TNF-α, IL-1β, and IL-6) in the serum of mice. Con, control. The data present the mean ± standard deviation (SD) (*n* = 6). Survival curves were calculated using the Kaplan-Meier method, and significance was determined by the log rank test. ns, no significance. One-way ANOVA followed by Tukey’s test was used to evaluate the statistical significance: ***, *P* < 0.001; ***, *P* < 0.0001. Different letters represent significant differences between different groups (*P* < 0.05).

A mouse model was established, and the mortality of the mice was observed and recorded (Fig. 1a). In the L-OHP group, two mice died from day 3 onward. In the 5-FU or CPT-11 group, only one mouse died on day 2 (Fig. 1b). Meanwhile, all mice remained alive on day 5 in the CF and control groups. 5-FU, CPT-11, and L-OHP effectively inhibited tumor growth (Fig. 1c and see Fig. S1 in the supplemental material). Contrary to the slight increase in body weight in the control and CF groups, body weight loss was observed in the 5-FU, CPT-11, and L-OHP groups (Fig. 1d). The weights of the mice in the control and CF groups increased by 6.66% and 5.68% (day 5 versus day 1), respectively. However, the weights decreased by 15.05%, 17.61%, and 27.55% in the 5-FU, CPT-11, and L-OHP groups, respectively. After the treatment with 5-FU, CPT-11, or L-OHP for 5 days, the diarrhea index, fecal occult blood (FOB) score, and disease activity index (DAI) were substantially higher than those of the control group. The mean diarrhea index values reached 1.86, 1.71, and 2.33 (Fig. 1e), the mean FOB scores reached 2.00, 2.00, and 2.67 (Fig. 1f), and the mean DAI values reached 4.86, 5.14, and 7.17 (Fig. 1g) for 5-FU, CPT-11, and L-OHP, respectively. No significant differences in diarrhea index, DAI, and FOB were observed between the CF and control groups.

### Secretion of inflammatory cytokines in the serum of mice treated with different chemotherapeutic drugs.

Administration of 5-FU, CPT-11, and L-OHP significantly enhanced the secretion of inflammatory cytokines, including IFN-γ, TNF-α, IL-1β, and IL-6, in the serum of mice compared with the cytokine levels of the control group (*P* < 0.05) (Fig. 1h). The cytokine levels were not significantly different between the CF and control groups (*P* > 0.05).

### Damage of different chemotherapeutic drugs to the liver, spleen, kidneys, and intestines.

**FIG 2:**
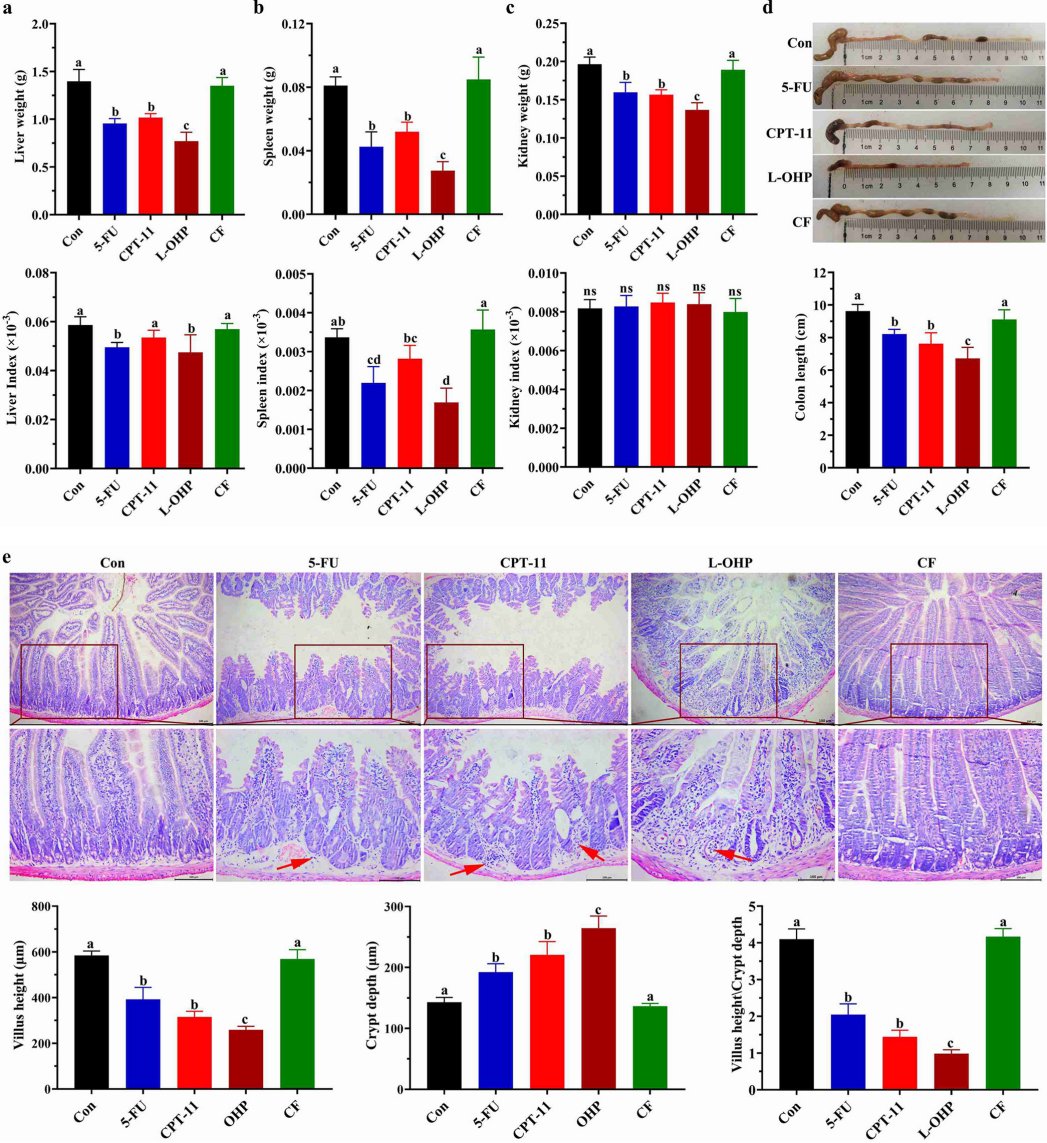
Different chemotherapeutic drugs caused organ lesions in mice. (a) Graphs of average liver weight and liver index in the different groups on day 5; (b) graphs of average spleen weight and spleen index of the different groups on day 5; (c) graphs of the average kidney weight and kidney index in the groups on day 5; (d) graphs of average colon length and a photograph for measuring and comparing colon length in the groups on day 5. (e) Hematoxylin and eosin (HE) staining of representative histological sections of the jejunum from the groups was performed (×200 magnification), and the villus height, crypt depth, and villus/crypt ratio were measured. One-way ANOVA followed by Tukey’s test was used to evaluate the statistical significance: different letters represent significant differences between different groups (*P* < 0.05).

The toxicological damage of different chemotherapeutic drugs to the liver, spleen, and kidneys was investigated. 5-FU, CPT-11, and L-OHP reduced the weight of the liver, spleen, and kidneys. The weight decreased to a higher degree in the L-OHP group than in the 5-FU or CPT-11 groups (*P* < 0.05) (Fig. 2a to c). Conversely, 5-FU and L-OHP reduced the liver and spleen index (tissues index = weight of tissues/weight of body) compared with those in the control group (*P* < 0.05) (Fig. 2a to c). Only the L-OHP group showed significantly histopathological damage to the liver and spleen (Fig. S2).

The intestinal injury induced by chemotherapeutic drugs was investigated. L-OHP, CPT-11, and 5-FU shortened the length of the colon but not CF (Fig. 2d). The results of histopathological tests showed that the intestines of the mice in the control and CF groups maintained an intact structure; conversely, obvious histological changes, such as shortening of the villi and the hyperplasia of the crypts of the jejunum (Fig. 2e), as well as inflammatory cell infiltration and large crypt area loss in the colon (Fig. S3), were observed in the 5-FU, CPT-11, and L-OHP groups. The histopathological results revealed that the L-OHP group induced a more serious intestinal damage than the 5-FU and CPT-11 groups did.

### Cellular proliferation and apoptosis of the jejunum under different chemotherapeutic drugs treatment.

**FIG 3:**
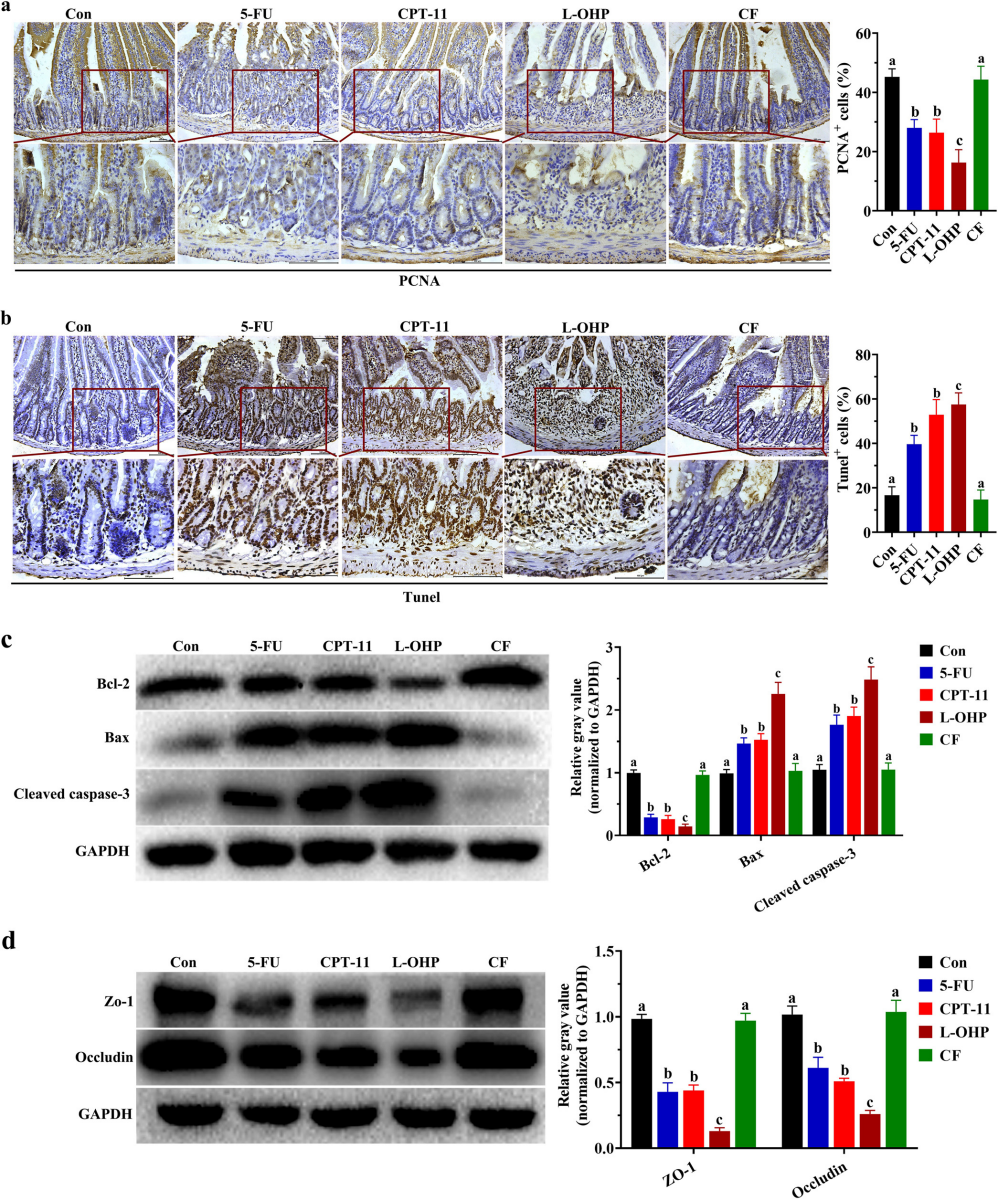
Effect of different chemotherapeutic drugs on apoptosis and proliferation. (a) Immunohistochemical (IHC) staining was used to detect the expression of PCNA and the average PCNA-positive cell proportions. (b) TUNEL assay for determining the DNA damage and the average apoptosis-positive cell proportions; (c and d) protein expression of cleaved-caspase 3, Bcl-2, and Bax (c) and ZO-1 and occludin (d). The protein production was normalized with GAPDH. One-way ANOVA followed by Tukey’s test was used to evaluate the statistical significance. The different letters represent significant differences between different groups (*P* < 0.05).

Chemotherapeutic drugs can induce the damage to the jejunum and colon, especially in the jejunum (18, 19). Therefore, the jejunum was used as the main tissue for the subsequent experiment. PCNA expression, an index of cell proliferation, was detected. The number of PCNA^+^ cells significantly decreased in the 5-FU, CPT-11, and the L-OHP groups (versus control, *P* < 0.05), and the decrease was dramatic in the L-OHP group (versus 5-FU and versus CPT-11, *P* < 0.05) (Fig. 3a). However, the number of PCNA^+^ cells in the CF group did not significantly decrease compared with that in the control group (*P* < 0.05). As expected, the terminal deoxynucleotidyltransferase-mediated dUTP-biotin nick end labeling (TUNEL) staining results were contrary to PCNA staining results (Fig. 3b).

To further study the apoptotic response in the jejunum, the levels of apoptosis-related molecules such as Bcl-2, Bax, and cleaved caspase-3 were detected. As shown in Fig. 3c, the administration of 5-FU, CPT-11, and L-OHP significantly reduced the protein level of Bcl-2 but increased the protein levels of Bax and cleaved caspase-3 (*P* < 0.05). Noticeably, these proteins changed more obviously in the L-OHP group than in the 5-FU or CPT-11 groups (*P* < 0.05). The protein levels of these molecules were not significantly different between the CF and control groups (*P* > 0.05).

### Expression of the jejunal mucosal barrier protein ZO-1 and occludin under different chemotherapeutic drugs.

The expression levels of ZO-1 and occludin, which are associated with the integrity and permeability of the jejunal mucosal barrier, were examined (Fig. 3d). The protein levels of ZO-1 and occludin were markedly decreased in the 5-FU, CPT-11, and L-OHP groups, and the decrease was more significant in the L-OHP group than the 5-FU or CPT-11 groups (*P* < 0.05). However, treatment with CF did not significantly change the ZO-1 and occludin expression levels compared with those of the control group (*P* > 0.05).

### Changes in gut microbiota under different chemotherapeutic drugs.

**FIG 4:**
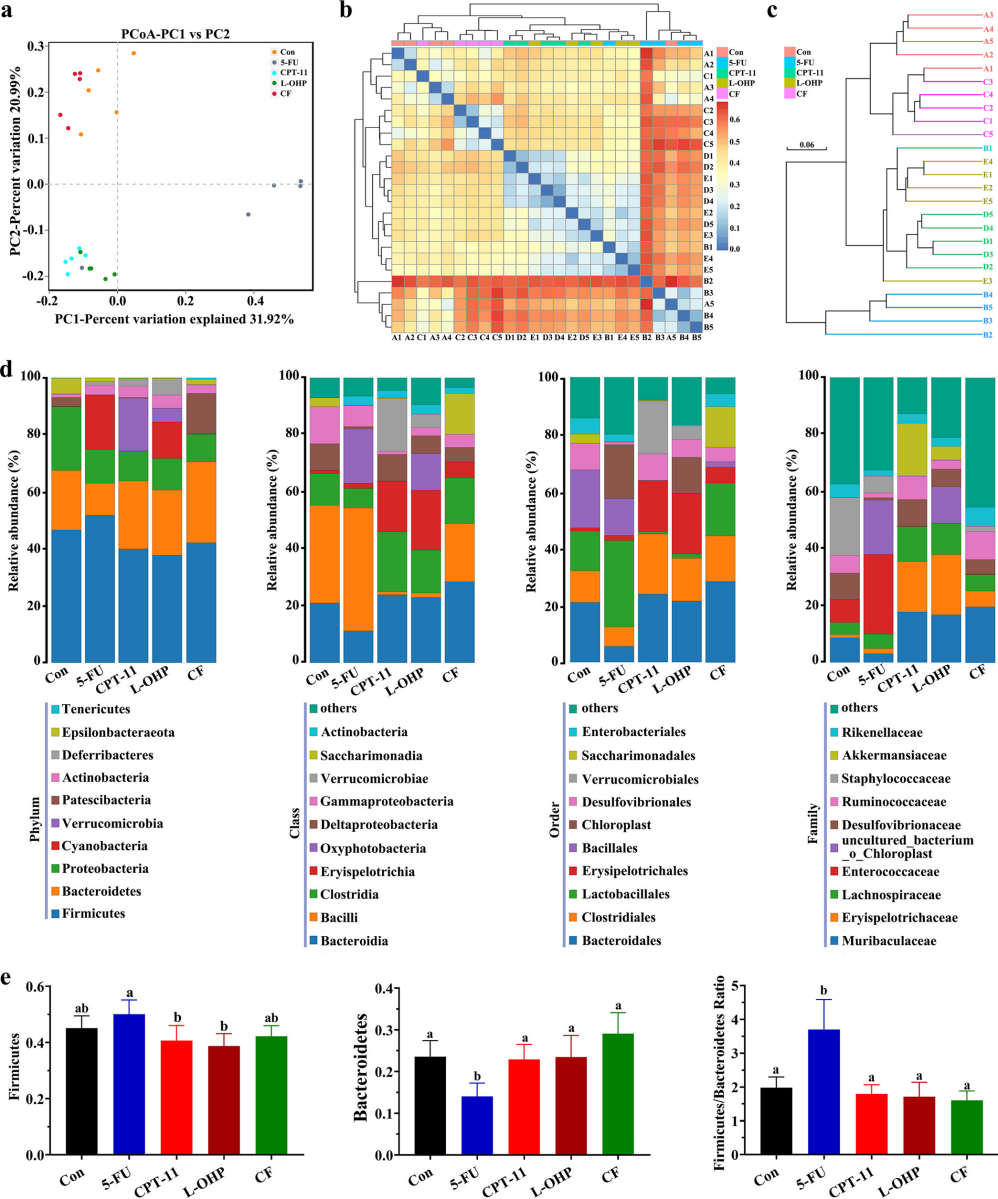
Effects of different chemotherapeutic drugs on the gut microbiota in mice. (a to c) Principal-coordinate analysis (PCoA) (a), heat map results of the weighted UniFrac distance (b), and arithmetic mean tree of the unweighted UniFrac distance (c) of microbial 16S rRNA sequences from the V3-V4 region in feces. (d) Average composition at the phylum, class, order, and family levels in the groups. (e) Graphs of gut microbiota composition were constructed using the levels of *Firmicutes* and *Bacteroidetes* as well as the *Firmicutes*/*Bacteroidetes* (F/B) ratio. One-way ANOVA followed by Tukey’s test was used to evaluate the statistical significance. The different letters represent significant differences between different groups (*P* < 0.05).

Intestinal microbiology serves as an important regulator of intestinal health. Disturbance in the gut microbiota can promote intestinal injury (20). Intestinal injury caused by chemotherapeutic drugs can be effectively alleviated by adjusting the gut microbiota (21). However, the patterns of gut microbiota disorders have not been compared among different colorectal cancer chemotherapeutic drugs; consequently, the treatment of chemotherapy-induced intestinal injury becomes difficult. In this study, we performed a systematic sequencing analysis by detecting the 16S rRNA gene in variable regions V3 to V4 to examine the changes in the gut microbiota from fecal samples in all groups (Fig. 4a to e).

The results of the binary-based principal-coordinate analysis (PCoA) showed the distinct clustering of the microbiota composition in each group (Fig. 4a and Fig. S4). Compared with the control group, no significant deviation was observed in the CF group, but not in the L-OHP, CPT-11, or 5-FU groups. The UniFrac-based weighted heat map (Fig. 4b) and the unweighted pair group method with the arithmetic mean tree (Fig. 4c) revealed that the microbial communities in the feces of the 5-FU, CPT-11, and L-OHP groups were significantly different from those in the control group. In addition, the samples of 5-FU and CPT-11 groups were almost separated into two independent groups, indicating that the changes in gut microbiota in the 5-FU and CPT-11 groups were nearly two different types, while the samples of the CPT-11 and L-OHP groups were clustered, suggesting that the changes in gut microbiota types were similar.

To profile the specific changes in the gut microbiota, we analyzed the relative abundance of the predominant taxa (top 10) (Fig. 4d and e). At the phylum level, *Proteobacteria* significantly decreased and *Actinobacteria* significantly increased in the 5-FU, CPT-11, and L-OHP groups (*P* < 0.05). *Patescibacteria* significantly decreased, and *Cyanobacteria* significantly increased in the 5-FU and L-OHP groups (*P* < 0.05). *Epsilonbacteraeota* decreased significantly, while *Verrucomicrobia* and *Deferribacteres* increased significantly in CPT-11 and L-OHP groups (*P* < 0.05 versus control). The *Firmicutes/Bacteroidetes* (F/B) ratio is used as a common parameter to assess the gut microbiota disorder in many diseases (22). As shown in Fig. 4d and e, the F/B ratio in the 5-FU group significantly increased compared with that in the control group (*P* < 0.05), indicating that the components of the gut microbiota were harmful. However, no significant change in the F/B ratio was found in the L-OHP, CPT-11, and CF groups.

**FIG 5:**
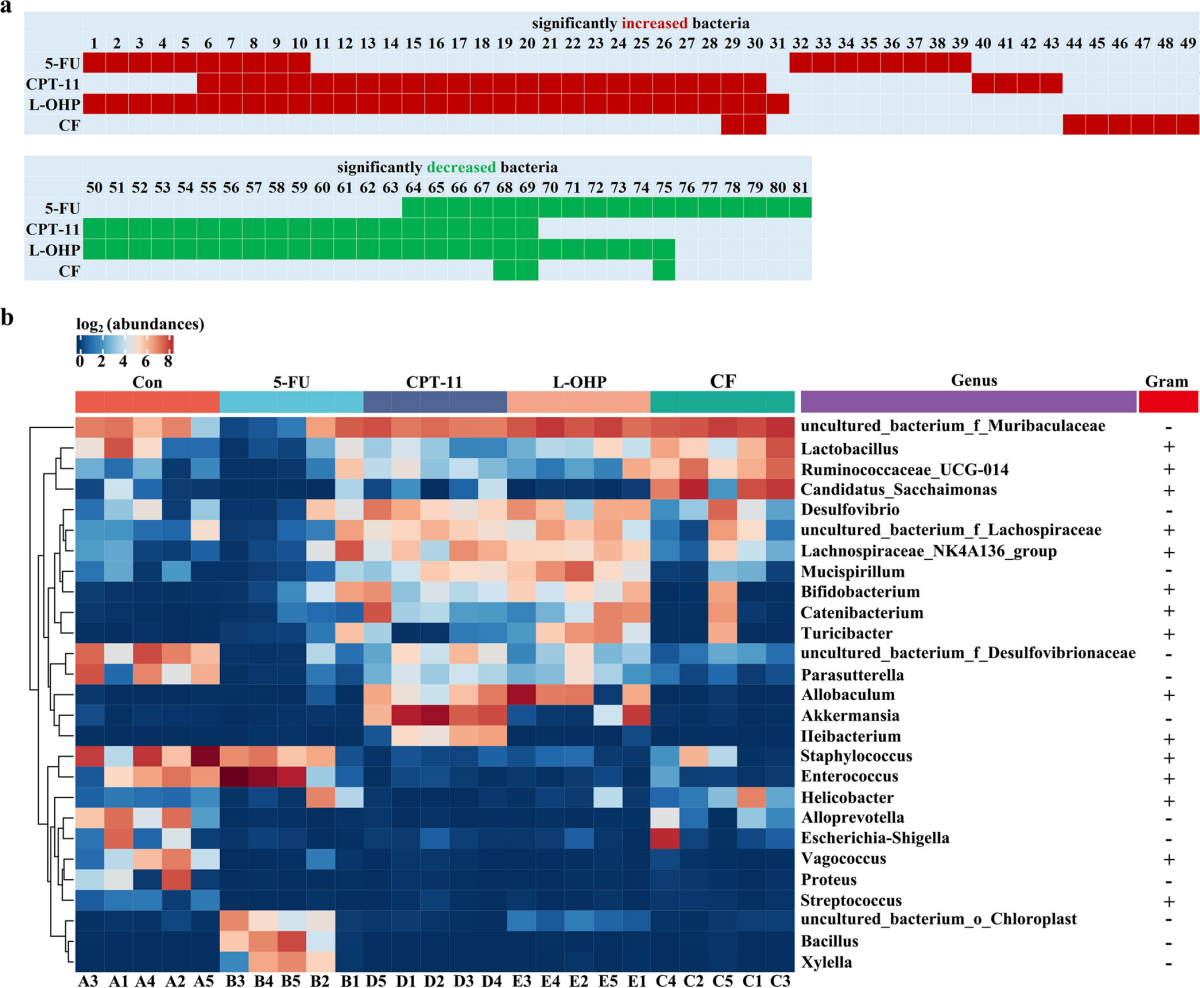
Effects of different chemotherapeutic drugs on microbiota taxonomic distributions in mice. (a) Significant changes in bacteria at all levels of phylum, class, order, family, and genus compared with those in the controls. (b) The heat map of 16S rRNA gene sequencing analysis of feces at the genus level showed 27 key species with significant differences.

To identify the bacterial taxonomic markers associated with intestinal injury and determine whether overlaps occurred among the microbial change patterns under different chemotherapeutic drugs, we constructed Fig. 5a, which was based on the results of linear discriminant analysis (LDA) effect size (LEfSe) analysis and covered the significant changes in bacteria at the taxonomic levels of phylum, class, order, family, and genus compared with those of the control (The names of the bacteria corresponding to each of the numbers are shown in Table 1.) CF slightly influenced the gut microbiota; only eight bacteria significantly increased, whereas three bacteria decreased (Fig. 5a and Table 1). Conversely, L-OHP had the greatest influence on the gut microbiota: specifically, 31 bacteria significantly increased, whereas 25 bacteria significantly decreased.

**TABLE 1:** Biological classifications and bacterium names corresponding to the numbers shown in Fig. 5a

No.	Biological classification and bacterium name	No.	Biological classification and bacterium name	No.	Biological classification and bacterium name
1	p__Cyanobacteria	28	g__Lachnospiraceae_NK4A136_group	55	o__Enterobacteriales
2	c__Oxyphotobacteria	29	f__Muribaculaceae	56	o__Lactobacillales
3	o__Chloroplast	30	g__uncultured_bacterium_f_Muribaculaceae	57	f__Enterobacteriaceae
4	f__uncultured_bacterium_o_Chloroplast	31	g__Turicibacter	58	f__Enterococcaceae
5	g__uncultured_bacterium_o_Chloroplast	32	o__Sphingobacteriales	59	f__Helicobacteraceae
6	p__Actinobacteria	33	o__Xanthomonadales	60	f__Staphylococcaceae
7	c__Actinobacteria	34	c__Alphaproteobacteria	61	g__Enterococcus
8	o__Bifidobacteriales	35	f__Bacillaceae	62	g__Helicobacter
9	f__Bifidobacteriaceae	36	f__Sphingobacteriaceae	63	g__Staphylococcus
10	g__Bifidobacterium	37	f__Xanthomonadaceae	64	f__Prevotellaceae
11	p__Deferribacteres	38	g__Bacillus	65	g__Proteus
12	p__Verrucomicrobia	39	g__Xylella	66	g__Escherichia_Shigella
13	c__Deferribacteres	40	c__Clostridia	67	g__Alloprevotella
14	c__Erysipelotrichia	41	o__Clostridiales	68	g__Vagococcus
15	c__Verrucomicrobiae	42	f__Lachnospiraceae	69	g__Streptococcus
16	o__Deferribacterales	43	g__Ileibacterium	70	p__Patescibacteria
17	o__Erysipelotrichales	44	p__Patescibacteria	71	c__Saccharimonadia
18	o__Verrucomicrobiales	45	c__Saccharimonadia	72	o__Saccharimonadales
19	f__Akkermansiaceae	46	o__Saccharimonadales	73	f__Saccharimonadaceae
20	f__Deferribacteraceae	47	f__Saccharimonadaceae	74	g__Candidatus_Saccharimonas
21	f__Erysipelotrichaceae	48	g__Candidatus_Saccharimonas	75	g__uncultured_bacterium_f_Desulfovibrionaceae
22	g__Akkermansia	49	g__Ruminococcaceae_UCG_014	76	c__Deltaproteobacteria
23	g__Allobaculum	50	p__Epsilonbacteraeota	77	o__Desulfovibrionales
24	g__Catenibacterium	51	c__Bacilli	78	f__Desulfovibrionaceae
25	g__Mucispirillum	52	c__Campylobacteria	79	f__Lactobacillaceae
26	g__Desulfovibrio	53	o__Bacillales	80	g__Lactobacillus
27	g__uncultured_bacterium_f_Lachnospiraceae	54	o__Campylobacterales	81	g__Parasutterella

The pattern of microbial changes in the L-OHP group intersected with those in the CPT-11 and 5-FU groups. Specifically, 29 bacteria significantly increased in the CPT-11 treatment, and 25 (86%) of them also increased in the L-OHP group. In addition, 19 bacteria significantly decreased under CPT-11 treatment, and all of them (100%) decreased in the L-OHP group. Interestingly, 18 bacteria significantly increased under 5-FU treatment, and 10 (55.6%) of them changed in the L-OHP group. Furthermore, 17 bacteria significantly decreased under 5-FU treatment, and 11 (64.7%) of them decreased in the L-OHP group (Fig. 5a). The patterns of microbial changes in 5-FU and CPT-11 almost differed (Fig. 5a).

A genus-level heat map with the relative abundance of 27 genera was constructed to show the change in the gut microbiota caused by 5-FU, CPT-11, L-OHP, or CF (Fig. 5b). The heat map of the relative abundance of microorganisms altered by the four chemotherapeutic drugs differed in gut bacterial composition compared with that of the control group at the genus level.

### Molecular mechanism of gut microbiota disorganization aggravates intestinal injury.

**FIG 6:**
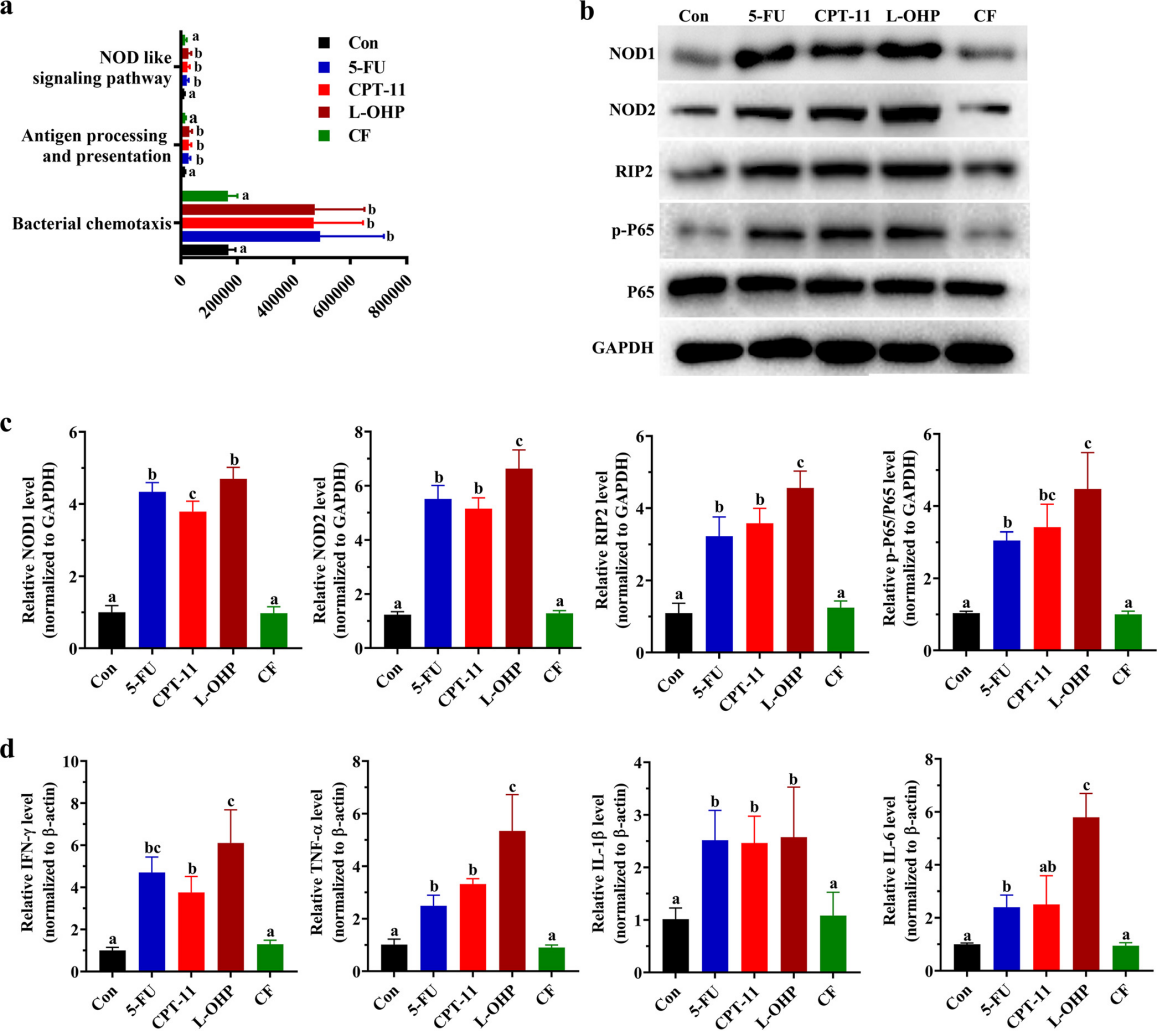
Effects of different chemotherapeutic drugs on the prediction and verification of potential metabolic functions of the gut microbiota in mice. (a) Microbial gene functions in the mice of different groups as indicated using the PICRUSt bioinformatic software package. The different letters represent significant differences between different groups (*P* < 0.05). (b to d) The effect of different chemotherapeutic drugs on NOD1/2/RIP2/NF-κB pathway proteins (b and c) and inflammatory cytokines (d). One-way ANOVA followed by Tukey’s test was used to evaluate the statistical significance. The different letters represent significant differences between different groups (*P* < 0.05).

We further used Phylogenetic Investigation of Communities by Reconstruction of Unobserved States (PICRUSt v.1.0) to predict the metabolic functions according to the changes in gut microbiota treated with four chemotherapeutic drugs. The relative levels of “bacterial chemotaxis,” “antigen processing and presentation,” and “NOD-like signaling pathway” were significantly higher in the 5-FU, CPT-11, and L-OHP groups than in the control and CF groups (Fig. 6a). Activation of the NOD-like pathway promotes the secretion of inflammatory cytokines and induces intestinal injury (23). Therefore, we hypothesized that the intestinal injury aggravation induced by the gut microbiota disorder under treatment with different chemotherapeutic drugs is associated with the NOD-like signaling pathway.

To test this hypothesis, we detected NOD1 and NOD2 gene expression at protein levels in jejunum tissues from different groups and found that NOD1/2 levels increased in 5-FU, CPT-11, and L-OHP groups (*P* < 0.05) (Fig. 6b and c). The activation of NOD1/2 can activate the NOD/RIP2/NF-κB (p65) signaling pathway to trigger inflammation (24). Under 5-FU, CPT-11, and L-OHP treatments, RIP2 and p-P65/P65 at the protein level were upregulated (Fig. 6b and c). Furthermore, we detected the inflammatory cytokines in jejunum tissues via quantitative PCR (qPCR). We found that administration of 5-FU, CPT-11, and L-OHP significantly enhanced the secretion of inflammatory cytokines, including IFN-γ, TNF-α, IL-1β, and IL-6 (*P* < 0.05), in the intestines compared with secretion in the control group (Fig. 6d).

### Correlation analysis of the biological indicators of the gut microbiota.

**FIG 7:**
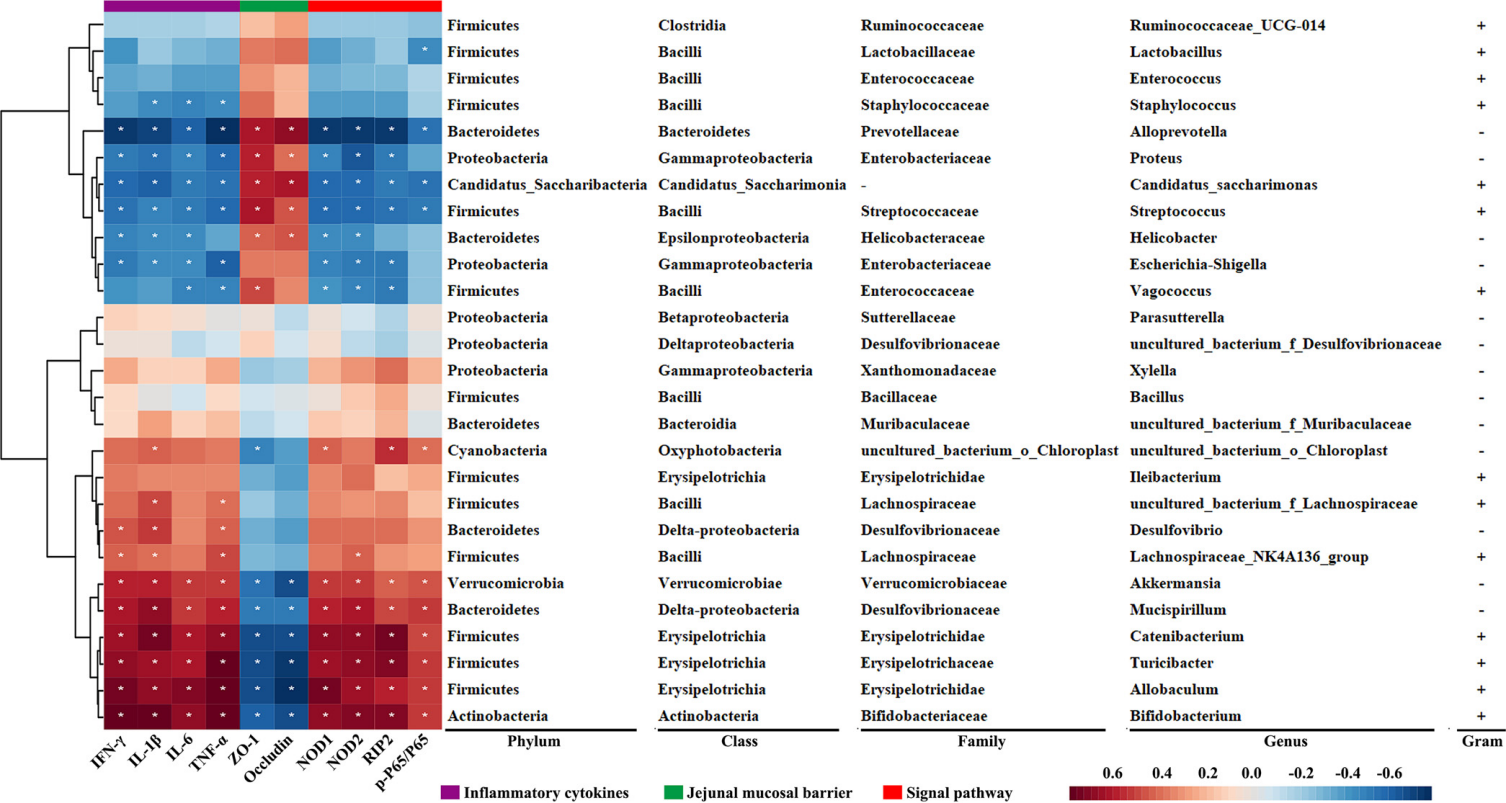
Correlation analysis of biological indicators related to the gut microbiota. The heat map shows the correlation coefficient between the gut microbiota and biological markers. A correlation coefficient of *R*^2^ > 0.25 indicates a moderate correlation and one of *R*^2^ > 0.36 indicates a strong correlation. An asterisk indicates the correlation significance (*P* < 0.05). Red represents a positive correlation, and blue represents a negative correlation. The darker the color, the stronger the correlation. Each row shows information on bacterial taxa (phylum, class, family, and genus).

According to the Pearson correction coefficient between 27 genera and 10 parameters (IFN-γ, IL-1β, Il-6, TNF-α, ZO-1, occludin, NOD1, NOD2, RIP2, and p-P65/P65), we created a correction matrix and found that 70.4% (19/27) of the 27 genera influenced by chemotherapeutic drugs were negatively or positively related to one or more parameters (Fig. 7). These data suggested that the gut microbiota plays an important role in chemotherapeutic drug-induced intestinal injury and is significantly related to the secretion of inflammatory cytokines, the integrity of the intestinal mucosa, and the NOD/RIP2/NF-κB pathway.

## DISCUSSION

Chemotherapeutic drugs influence not only tumor cells but also normal cells. The use of chemotherapeutic drugs is usually accompanied by mucosal lesions because of the overproduction of proinflammatory cytokines and apoptosis of intestinal epithelial cells (25). The destruction of the intestines induced by chemotherapeutic drugs causes various side effects, such as abdominal pain, diarrhea, fecal blood, and even death in severe cases (9, 10). Therefore, clarifying the level of different chemotherapeutic drug-induced intestinal injury and the main mechanism can help develop targeted detoxification therapy and increase clinical chemotherapeutic application, which is meaningful for the treatment of colon cancer.

Gut microbiota disorder aggravates intestinal injury. and the two promote each other's deterioration (26, 27). Although chemotherapeutic drugs, such as 5-FU, CPT-11 and L-OHP, can induce intestinal injury (28), studies have yet to compare the differences between the intestinal injury degree and gut microbiota disorder pattern among them, which form the FOLFIRI (folinic acid, 5-FU, and CPT-11) and FOLFOX colorectal cancer chemotherapy regimens (29). Our study excluded the effects of different operators, seasonal climates, batches of mice, and mice from different environments, which allowed us to obtain accurate results for 5-FU-, CPT-11-, L-OHP-, and CF-induced intestinal injury and gut microbiota disorder in BALB/c mice. The comparison of these results allows the development of new ideas and a scientific theory for reducing the intestinal side effects of colorectal cancer chemotherapy.

5-FU, an S-phase-specific anticancer drug, causes intestinal injury in 50% to 80% of patients (30). L-OHP, a third-generation platinum antitumor compound, can form inter- or intrastrand cross-links in DNA to prevent DNA replication and transcription, resulting in apoptosis. The common adverse reaction of L-OHP is colon toxicity, and the diarrhea-inducing dose of L-OHP overlaps its lethal dose (5). Chemotherapy with CPT-11 also induces intestinal mucositis with the increased expression of proinflammatory cytokines regulated by NODs (31). CF alone does not inhibit tumor growth, but in combination with 5-FU, it increases the antitumor effect of 5-FU (32).

In this study, mice under 5-FU or CPT-11 treatment displayed moderate weight loss, diarrhea, and fecal blood symptoms. L-OHP treatment caused a more severe damage than 5-FU and CPT-11 did (Fig. 1). 5-FU, CPT-11, and L-OHP could induce colon shortening and significantly cause colon and jejunum injury, and the most severe intestinal injury was caused by L-OHP. In addition, only L-OHP could cause significant histopathological injury to the liver and spleen. Therefore, morphological observations showed that L-OHP had the most severe side effects and caused the most serious intestinal injury among the main colorectal cancer chemotherapeutic drugs (Fig. 2).

Caspase-3 is a key executor of apoptosis, which can be cleaved to its activated form and induces cell apoptosis. The movement of Bax from the cytosol to the mitochondria induces cytochrome *c* release and caspase-3 activation, which can be inhibited by Bcl-2 (33). The administration of 5-FU, CPT-11, and L-OHP increased the protein levels of Bax and cleaved caspase-3 but decreased the protein level of Bcl-2. However, the protein levels of these molecules in the CF group were not significantly different from those in the control group (Fig. 3). As the first line of immune defense, the intestinal epithelial barrier is crucial for protecting the host against invasive pathogenic bacteria. Once the integrity of the intestinal barrier is lost, bacteria and toxic substances can penetrate the intestinal wall and trigger the feedback cycle (34). We found that 5-FU, CPT-11, and L-OHP significantly decreased the expression of ZO-1 and occludin (Fig. 3), which are markers of mucosal barrier integrity in the jejunum (35).

We analyzed the composition of the intestinal microbiota in the feces. PcoA results revealed that despite access to the same food, the microbial communities under 5-FU, CPT-11, or L-OHP treatment varied considerably. The results of β-diversity heat map and ClusterTree analyses were in accordance with those of PCoA and showed that the microbial communities under L-OHP treatment were more similar to those under CPT-11 than under 5-FU (Fig. 4). The F/B ratio was used as a common parameter to assess the degree of intestinal microbiota disturbance (36). In this study, at the phylum level, the F/B ratio was increased in the 5-FU group (*P* < 0.05 versus control), while no significant difference was observed in CPT-11, L-OHP, and CF groups (*P* > 0.05 versus control). The change in the F/B ratio confirmed that the type of gut microbiota change induced by CPT-11 and L-OHP was different from that caused by 5-FU. The relative abundance of microbiota at the class, order, and family levels was also consistent with this phenomenon (Fig. 4).

To provide guidelines for the targeted treatment of chemotherapy-induced intestinal injury, we carefully compared the change patterns of the 5-FU, CPT-11, L-OHP, and CF groups. LEfSe was used to identify microbial biomarkers. Furthermore, 86% (25/29) of the increased bacteria and 100% (19/19) of the decreased bacteria under CPT-11 treatment appeared in the change pattern under L-OHP treatment. However, only 17.2% (5/29) of the increased bacteria and 26.3% (5/19) of the decreased bacteria under CPT-11 treatment appeared in the microbial change pattern of 5-FU. In the microbial change pattern under 5-FU treatment, 55.6% (10/18) of the increased bacteria and 64.7% (11/17) of the decreased bacteria appeared in the L-OHP group (Fig. 5). In combination with the intestinal injury test, L-OHP caused more severe injury than either 5-FU or CPT-11 did (Fig. 1 and 2). Therefore, we hypothesized that L-OHP caused the most severe intestinal injury because of the most extensive microbial change pattern, and the pattern overlapped those of CPT-11 and 5-FU.

On the basis of these results, we further compared the influence of 5-FU, CPT-11, L-OHP, and CF on the genus-level taxonomic distributions of the microbial communities in feces (Fig. 5). After 5-FU treatment, the abundance of *Lactobacillus*, *Parasutterella*, *Alloprevotella*, Escherichia*-Shigella*, *Vagococcus*, Proteus, uncultured_bacterium_f_Desulfovibrionaceae, and “*Candidatus* Saccharimonas” significantly reduced, while the abundance of *Bifidobacterium*, uncultured_bacterium_o_Chloroplast, *Bacillus*, and *Xylella* significantly increased. Gram-positive *Lactobacillus* and *Parasutterella* are beneficial bacteria, and *Parasutterella* is related to short-chain fatty acid (SCFA) production (37). Gram-negative *Alloprevotella* is also an SCFA-producing bacterium (38). Escherichia*-Shigella* is associated with inflammation in acute necrotizing pancreatitis (ANP) (39) and intestinal inflammation (40) models. The abundance of intestinal *Vagococcus* in healthy individuals is significantly higher than that in patients with cancer (41). Proteus species are usually considered commensals in the gut and recognized clinically as putative gastrointestinal pathogens (42). The abundance of “*Candidatus* Saccharimonas” decreases significantly in the ANP model (39, 43) but increases significantly in the colitis-associated carcinogenesis model (44). *Bifidobacterium* is a probiotic that protects the neonatal intestine against necrotizing enterocolitis (45). Some *Bacillus* species, such as B. cereus, B. anthracis, and B. mycoides, are human pathogens (46). *Xylella* is a common pathogen in plants (47), but its abundance significantly increased with 5-FU intervention.

After CPT-11 treatment, the abundance of *Vagococcus*, Proteus, Escherichia*-Shigella*, *Alloprevotella*, *Enterococcus*, *Helicobacter*, and Staphylococcus significantly decreased, whereas the abundance of *Akkermansia*, *Allobaculum*, *Ileibacterium*, *Catenibacterium*, *Bifidobacterium*, *Desulfovibrio*, Lachnospiraceae_NK4A136 group, *Mucispirillum*, uncultured_bacterium_Lachnospiraceae, and uncultured_bacterium_Muribaculaceae significantly increased. *Enterococcus* is a common pathogen of prostatitis (48), but it is not highly virulent. *Helicobacter* is a potent driver of colonic T cells and positively correlated with body weight (38). The majority of Staphylococcus species are nonpathogenic bacteria that can cause diseases (49). *Akkermansia* is considered a probiotic in most studies, but the presence of excessive *Akkermansia* can cause intestinal injury (50). The excessive abundance of *Catenibacterium*, *Bifidobacterium*, and *Allobaculum* has a significantly positive relationship with serum inflammatory factors and may aggravate the severity of colitis (51, 52). *Ileibacterium* is a newly identified group of the genus *Allobaculum* (53). *Desulfovibrio* and Lachnospiraceae_NK4A136 are harmful bacteria associated with a high-fat diet and chronic inflammation (54, 55). *Mucispirillum* inhabits the mucus layer over colonocytes and is associated with the early disruption of the colonic surface mucus layer (56).

The gut microbiota under L-OHP treatment almost significantly varied in the microbial change pattern of CPT-11 or 5-FU group, except *Turicibacter*, which aggravates the severity of colitis (51). The abundance of *Vagococcus*, Proteus, Escherichia*-Shigella*, and *Alloprevotella* decreased in the L-OHP, CPT-11, and 5-FU groups. *Enterococcus*, *Helicobacter*, and Staphylococcus levels decreased in the L-OHP and CPT-11 groups. “*Candidatus* Saccharimonas” and uncultured bacterium_f_Desulfovibrionaceae decreased in the L-OHP and 5-FU groups. *Bifidobacterium* increased in the L-OHP, CPT-11, and 5-FU groups. *Desulfovibrio*, Lachnospiraceae_NK4A136_group, *Mucispirillum*, *Akkermansia*, *Allobaculum*, *Catenibacterium*, uncultured_bacterium_Lachnospiraceae, and uncultured_bacterium_Muribaculaceae increased in the L-OHP and CPT-11 groups. uncultured_bacterium_o_Chloroplast increased in the L-OHP and 5-FU groups.

Differences in the functional genes of the gut microbiota in metabolic pathways between different chemotherapeutic drug groups were predicted by PICRUSt. The influence of the gut microbiota on the host cellular processes, organismal systems, and environmental information processing may lead to the occurrence of diseases. We found that the influence of 5-FU, CPT-11, and L-OHP on the gut microbiota was found to be related to “bacterial chemotaxis,” “antigen processing and presentation,” and “NOD-like signaling pathway” (Fig. 6). Bacterial chemotaxis is a phenomenon through which bacteria direct their movements according to certain chemical stimulants in their environment (57), and it helps harmful bacteria to colonize the intestinal mucosa and promote intestinal injury (58). NODs mediate the innate immune system and regulate the expression of proinflammatory cytokines (59, 60). The NOD-like signaling pathway activated by intestinal mucosal injury may induce immune responses and aggravate injury severity (61). The NOD/RIP2/NF-κB signaling pathway is closely associated with oxidative stress and inflammatory responses (62, 63), and NF-κB can alter the expression of inflammatory cytokines such as IFN-γ, TNF-α, IL-1β, and IL-6 to cause inflammatory injury in the intestinal barrier (64). These results suggested that chemotherapeutic drugs might further activate the expression of proinflammatory cytokines to promote intestinal injury by activating the NODs/RIP2/NF-κB signaling pathway. Furthermore, 5-FU intervention has been reported to activate the NOD-like signaling pathway (65). The protein expression levels of NOD1, NOD2, RIP2, and p-NF-κB/NF-κB and the secretion level of proinflammatory cytokines in the intestines confirmed this hypothesis (Fig. 6). Furthermore, on the basis of the correlation analysis of intestinal injury biological indicators and the gut microbiota, we found that 19 of the 27 genera (70.3%) were associated with at least one parameter, and 13 of the 27 genera (48%) were associated with at least six parameters (Fig. 7).

**FIG 8:**
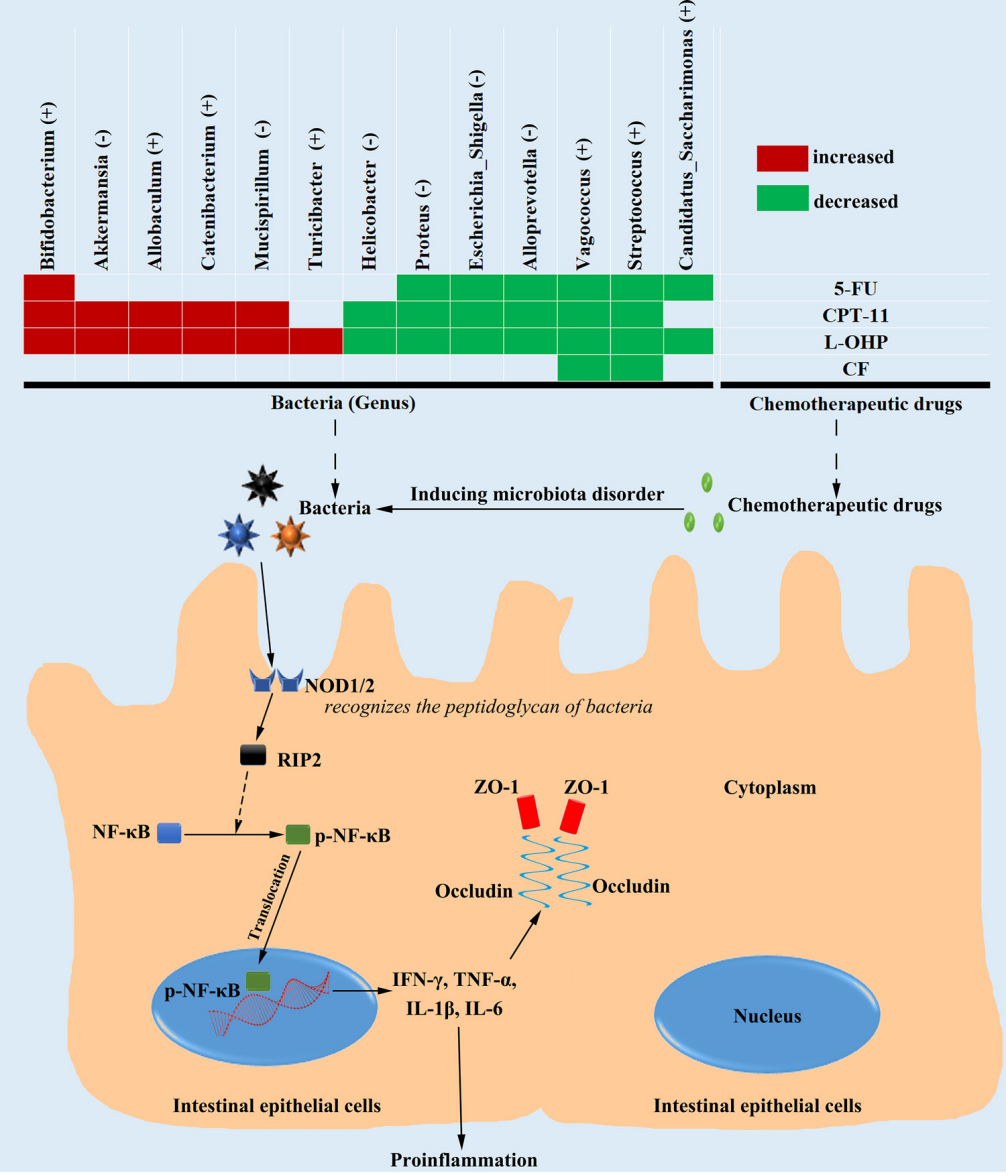
Mechanisms of different degrees of intestinal injury induced by chemotherapeutic drugs (5-FU, CPT-11, L-OHP, and CF) for colorectal cancer.

In conclusion, 5-FU, CPT-11, L-OHP, and CF, chemotherapeutic drugs for colorectal cancer, exhibited different toxicities to intestinal injury and gut microbiota, and L-OHP induced the most severe damage. The microbial change pattern under L-OHP treatment significantly overlapped the changes in the 5-FU and CPT-11 groups, but the microbial change pattern did not overlap between 5-FU and CPT-11. The NOD/RIP2/NF-κB signaling pathway was also mostly activated under L-OHP treatment. Thirteen genera of particular interest were *Bifidobacterium*, *Akkermansia*, *Allobaculum*, *Catenibacterium*, *Mucispirillum*, *Turicibacter*, *Helicobacter*, Proteus, Escherichia*-Shigella*, *Alloprevotealla*, *Vagococcus*, Streptococcus, and “*Candidatus* Saccharimonas,” which showed different abundances in the four drug groups and were most likely correlated with the mechanism of intestinal injury. These results might explain why different chemotherapeutic drugs induced different levels of intestinal injury (Fig. 8). We suggested that using bacteriostatic agents should be targeted to different colorectal cancer chemotherapeutic drugs. The use of cocktail therapy (e.g., FOLFOX or FOLFIRI) varies from person to person, and the proportion of different chemotherapeutic agents in the composition of the intervention varies (66). If L-OHP is the main drug in the cocktail therapy treatment, according to the conclusions in this article, it could be targeted primarily at the microbiota pattern changes caused by L-OHP. Use of another cocktail therapy (e.g., with 5-FU plus CPT-11 as the main drugs) needs to consider both 5-FU- and CPT-11-induced microbiota pattern changes because the changes of gut microbiota induced by 5-FU and CPT-11 were nearly two different types. We provided new ideas and new research directions for the targeted treatment of intestinal injury caused by colorectal cancer chemotherapy.

## MATERIALS AND METHODS

### Cell culture.

CT-26 cells (Cell Bank of the Chinese Academy of Sciences, Shanghai, People’s Republic of China) were cultured in RPMI 1640 medium (C11875500BT; Life Technologies, Carlsbad, CA, USA) containing 10% (vol/vol) fetal bovine serum (10099141; Life Technologies) and 1% penicillin-streptomycin (SV30010; Life Technologies) at 37°C in a humidified incubator with 5% CO_2_.

### Construction of an intestinal injury model induced by chemotherapeutic drugs in tumor-bearing mice.

Four-week-old male BALB/c mice weighing 20 to 24 g were obtained from the SLAC Laboratory Animal Technology Co., Ltd. (Shanghai, People’s Republic of China). The mice were maintained under specific-pathogen-free conditions (12-h/12-h light/dark cycle, 23°C to 25°C, 45% to 55% relative humidity) and given *ad libitum* access to autoclaved food and water. After 1 week of preexperimental adaptation, all mice were subcutaneously injected with 4 × 10^6^ CT-26 cells in the right armpit. Tumor-bearing mice were randomly divided into five experimental groups (*n* = 8): control, 5-FU, CPT-11, L-OHP, and CF. According to previous studies (18) and our investigation of preproject modeling conditions, the chemotherapeutic clinical equivalent dose was used as the intervention dose to induce chemotherapy-induced intestinal injury (Fig. 1a). The mice in different groups were intraperitoneally administered 200 μL of saline (control), 5-FU (100 mg/kg) (R050245; RHAWN, Shanghai, People’s Republic of China), CPT-11 (85 mg/kg) (B21490; Shanghai Yuanye Bio-Technology Co., Ltd., Shanghai, People’s Republic of China), L-OHP (20 mg/kg) (R002057; RHAWN, Shanghai, People’s Republic of China), and CF (100 mg/kg) (R054174; RHAWN, Shanghai, People’s Republic of China) for 5 consecutive days.

Body weight, diarrhea index, FOB score, and number of mortalities were recorded daily. The severity of body weight loss, diarrhea index, and FOB were assessed on the basis of Table S1 in the supplemental material. FOB was detected using a BaSO OB kit (BA2020B; BaSO, Zhuhai, People’s Republic of China). Then, the DAI (FOB + diarrhea index + body weight loss index) was calculated. All mice were anesthetized with sodium pentobarbital and euthanized after 16 h of fasting on day 6. Next, the jejunum and colon were cut into 1-cm sections, and one tissue was fixed in 4% paraformaldehyde for histological or immunohistochemical evaluation. The other jejunal tissues for transcriptome (RNA-seq) analysis, Western blotting, and qPCR were frozen at −80°C. Sera were collected for the detection of proinflammatory cytokines.

All animal-related protocols of this study conformed to the rules of the Animal Experimental Ethics Committee of Fujian University of Traditional Chinese Medicine and were performed in accordance with the National Institutes of Health *Guide for the Care and Use of Laboratory Animals* (67).

### Histological analysis of the intestinal injury.

The jejunal and colonic tissues were collected, fixed in 4% paraformaldehyde, and embedded in paraffin. They were then cut into 4-μm-thick sections to prepare slides and stained with hematoxylin and eosin (HE) (Solarbio Science, Beijing, People’s Republic of China). Pathological changes were observed under a light microscope (Leica DM4000B; Leica Co., Germany). The villus height and crypt depth of the jejunum and colon were measured in at least 10 clear longitudinal sections.

### Enzyme-linked immunosorbent assay.

The levels of IFN-γ, TNF-α, IL-1β, IL-6, and lipopolysaccharide (LPS) in the serum of each mouse were detected according to the enzyme-linked immunosorbent assay (ELISA) kit protocol (Meimian, Jiangsu, People’s Republic of China).

### Immunohistochemical evaluation.

PCNA expression was detected using immunohistochemical (IHC) analysis. The intestinal slides were deparaffinized and rehydrated. Endogenous peroxidase was blocked with 3% H_2_O_2_. Sections were incubated with primary antibodies at 4°C overnight, anti-PCNA antibody (1:200) (Sanying Biotechnology, Hubei, People’s Republic of China), and secondary antibodies (1:200) (Sanying Biotechnology, Hubei, People’s Republic of China), and stained with 3,3′-diaminobenzidine (DAB) and hematoxylin. Sections were observed under a light microscope (Leica). The integrated optical density of the sections positively stained for the proteins was measured using ImageJ version 1.8.0.

### Apoptosis assessment (TUNEL staining).

For apoptosis assessment, the slides of the jejunal sections were processed for the TUNEL assay (MK1025; Boster, Hubei, People’s Republic of China). Images were taken using a microscope (Leica) at ×200 and ×400 magnification.

### Western blot analysis.

Jejunum tissues were harvested and total protein was extracted using radioimmunoprecipitation assay lysis buffer containing proteinase inhibitor (Beyotime, Haimen, People’s Republic of China). Total protein content was determined using a bicinchoninic acid (BCA) protein assay (Beyotime). After quantification, protein samples were separated through SDS-PAGE, transferred to polyvinylidene difluoride (PVDF) membranes, and incubated with one primary antibody (Table S2) and horseradish peroxidase (HRP)-conjugated secondary antibodies. Chemiluminescent detection was conducted using the ChemiDoc XRS+ imaging system (Bio-Rad Laboratories, Inc., Hercules, CA, USA) according to the manufacturer’s instructions. ImageJ software was used to determine protein expression levels.

### qPCR.

Total RNA was extracted from jejunum tissues by using RNA isolation reagent (TaKaRa Biotechnology, Dalian, Liaoning, People’s Republic of China) according to the manufacturer’s instructions for animal tissue. Subsequently, the total RNA was reverse transcribed to cDNA by using the Prime Script RT reagent kit (TaKaRa Biotechnology). qPCR was performed using the SYBR Premix *Ex Taq* II kit (TaKaRa Biotechnology, Dalian, Liaoning, People’s Republic of China) on ABI 7500 sequence detection system software version 1.2.3 (Applied Biosystems, CA, USA). The thermocycling conditions were as follows: 50°C for 2 min, 95°C for 10 min, and 40 cycles of 95°C for 15 s and 60°C for 1 min. β-Actin was used as an endogenous control. The threshold cycle (2^−ΔΔ^*^CT^*) method was used to calculate the Toll-like receptor 4 (TLR4) mRNA levels.

### Gut microbiota analysis.

**(i) DNA extraction.** Feces were collected from five mice per group and immediately stored at −80°C. DNA from feces was extracted using MN NucleoSpin 96 Soil (MN; 740787) according to the manufacturer’s instructions.

**(ii) Sequence analysis: hypervariable region V3rer instructions.** The DNA concentration was determined using a NanoDrop 2000 spectrophotometer (Thermo Scientific) with primers 341F (CCTAYGGGRBGCASCAG) and 806R (GGACTACNNGGGTATCTAAT). Library construction and amplicon sequencing were performed using Genomics BioScience. A paired-end library (insert size of 460 bp for each sample) was constructed using the TruSeq Nano DNA library prep kit (Illumina), and high-throughput sequencing was performed on an Illumina HiSeq 2500 sequencer with a HiSeq Rapid v2 reagent kit (Illumina). The sequences of 2 × 250-bp paired-end reads were produced from the sequencer following the manufacturer’s instructions. The paired reads were merged into amplicon sequences by using FLASH v1.2.7, and these amplicon sequences were checked for the existence of the primers, duplicates were removed, and short sequences and chimeric reads were filtered out to generate effective reads. Effective reads were analyzed to generate operational taxonomic units (OTUs). Further 16S rRNA gene analysis (picking and taxonomic assignment) and data visualization were conducted using Quantitative Insights into Microbial Ecology (QIIME) version 1.8.0 with the Silva 16S rRNA Taxonomy Database (release 132). Taxonomy (i.e., phyla and OTUs) was analyzed using one-way analysis of variance (ANOVA).

**(iii) Microbial analyses.** The PCoA of the unweighted UniFrac distance matrix was displayed as β diversity to clarify the diversity of intestinal microbiota composition among all experimental groups (control, 5-FU, CPT-11, L-OHP, and CF). Weighted UniFrac distance matrices were computed to detect global variations in the composition of microbial communities at the phylum and genus levels through one-way ANOVA. Microbiota-based biomarker discoveries were performed using LEfSe. The relative abundance of each biomarker taxon across all samples is shown with straight and dotted lines that plot the means and medians, respectively, in each group. Color codes indicate the groups, and letters indicate the taxa that contribute to the uniqueness of the corresponding groups at an LDA value of >4.0.

**(iv) Functional capacity of the microbiota.** PICRUSt was used to predict the 16S rRNA-based high-throughput sequencing data for functional features from the phylogenetic information with an estimated accuracy of 0.8. The cluster of orthologous group database was obtained through the Green-gene ID corresponding to ea©OTU in the EGGNOG database (“evolutionary genealogy of genes: non-supervised orthologous groups”). The predicted functional composition profiles were then collapsed into class 3 of the Kyoto Encyclopedia of Genes and Genomes (KEGG) database pathways. The correlations between 27 genera and the intestinal mucosal barrier (ZO-1 and occludin), inflammatory cytokines (IFN-γ, TNF-α, IL-1β, and IL-6), and PI3K/AKT/NF-κB signaling pathway-related genes in mice treated with different chemotherapeutic drugs were determined by redundancy analysis/canonical correlation analysis (RDA/CCA) and the related heat map.

### Statistical analysis.

Statistical analysis was performed using SPSS software (version 23.0; SPSS, Inc., Chicago, IL, USA). Data are presented as mean ± standard deviation (SD). Survival curves were calculated using the Kaplan-Meier method, and significance was determined by the log-rank test. One-way ANOVA followed by Tukey’s *post hoc* test was used for multiple comparisons. Pearson’s correlation was used to determine the relationships between the parameters. Statistical significance was set at a *P* value of *<*0.05.

### Data availability.

The sequenced data reported in this article have been submitted to the NCBI Sequence Read Archive (SRA) under accession no. PRJNA832531.
