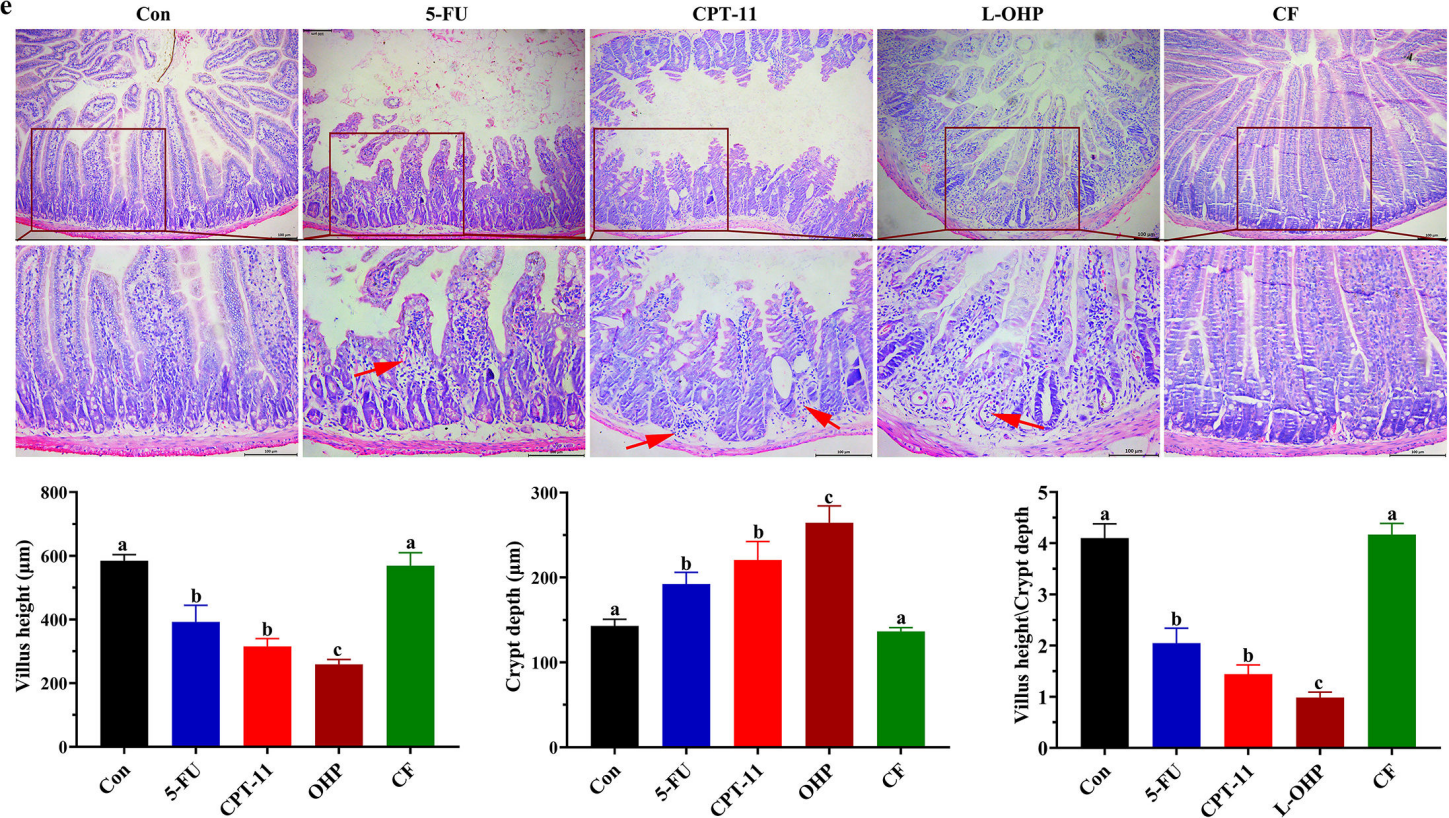# Correction for Huang et al., “Chemotherapeutic Drugs Induce Different Gut Microbiota Disorder Pattern and NOD/RIP2/NF-κB Signaling Pathway Activation That Lead to Different Degrees of Intestinal Injury”

**DOI:** 10.1128/spectrum.00112-24

**Published:** 2024-03-26

**Authors:** Bin Huang, Mengxuan Gui, Zhuona Ni, Yanbin He, Jinyan Zhao, Jun Peng, Jiumao Lin

## AUTHOR CORRECTION

Volume 10, no. 6, e01677-22, 2022, https://doi.org/10.1128/spectrum.01677-22. Panel e of Fig. 2 should appear as shown in this correction. The H&E picture for the 5-FU group mistakenly features a sample image of the CPT-11 group owing to our negligence while adjusting the layout of the final picture version. The revisions do not affect any results and conclusions of the work. We sincerely apologize for any inconvenience caused to the journal and readers.

**Fig 2:**