# Supplementary material for: Chemotherapeutic Drugs Induce Different Gut Microbiota Disorder Pattern and NOD/RIP2/NF-κB Signaling Pathway Activation That Lead to Different Degrees of Intestinal Injury
Source: Microbiol Spectr. 2022 Oct 12;10(6):e01677-22. doi: 10.1128/spectrum.01677-22 (PMC9769542; doi:10.1128/spectrum.01677-22)
Supplement: Supplemental file 1 — Fig. S1 to S4 and Tables S1 and S2. Download spectrum.01677-22-s0001.pdf, PDF file, 3.3 MB [file spectrum.01677-22-s0001.pdf]

Supplementary Figures

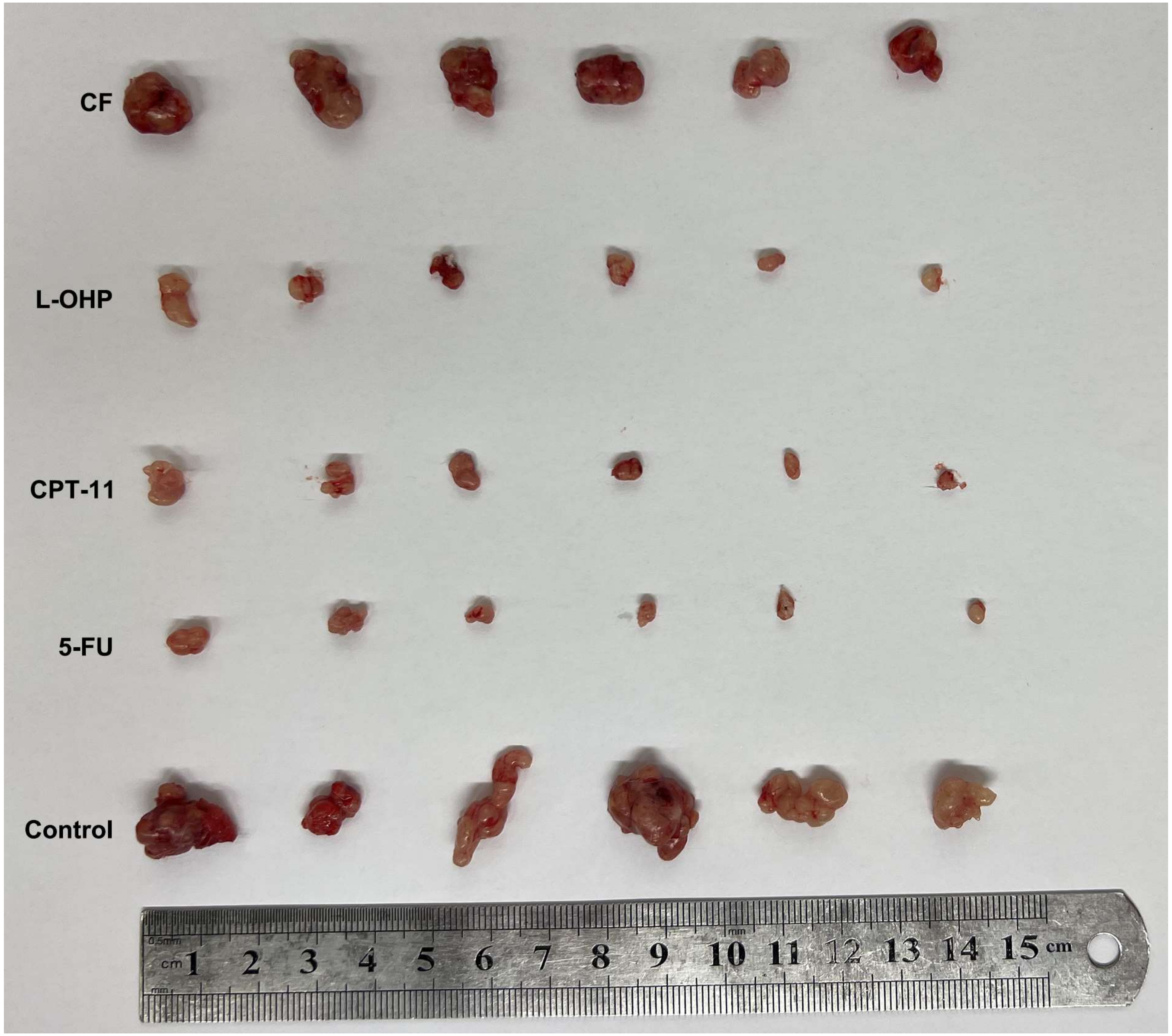

FIG S1 The effect of chemotherapeutic drugs (5-FU, CPT-11, L-OHP and CF) on inhibiting the growth of subcutaneous xenograft tumors.

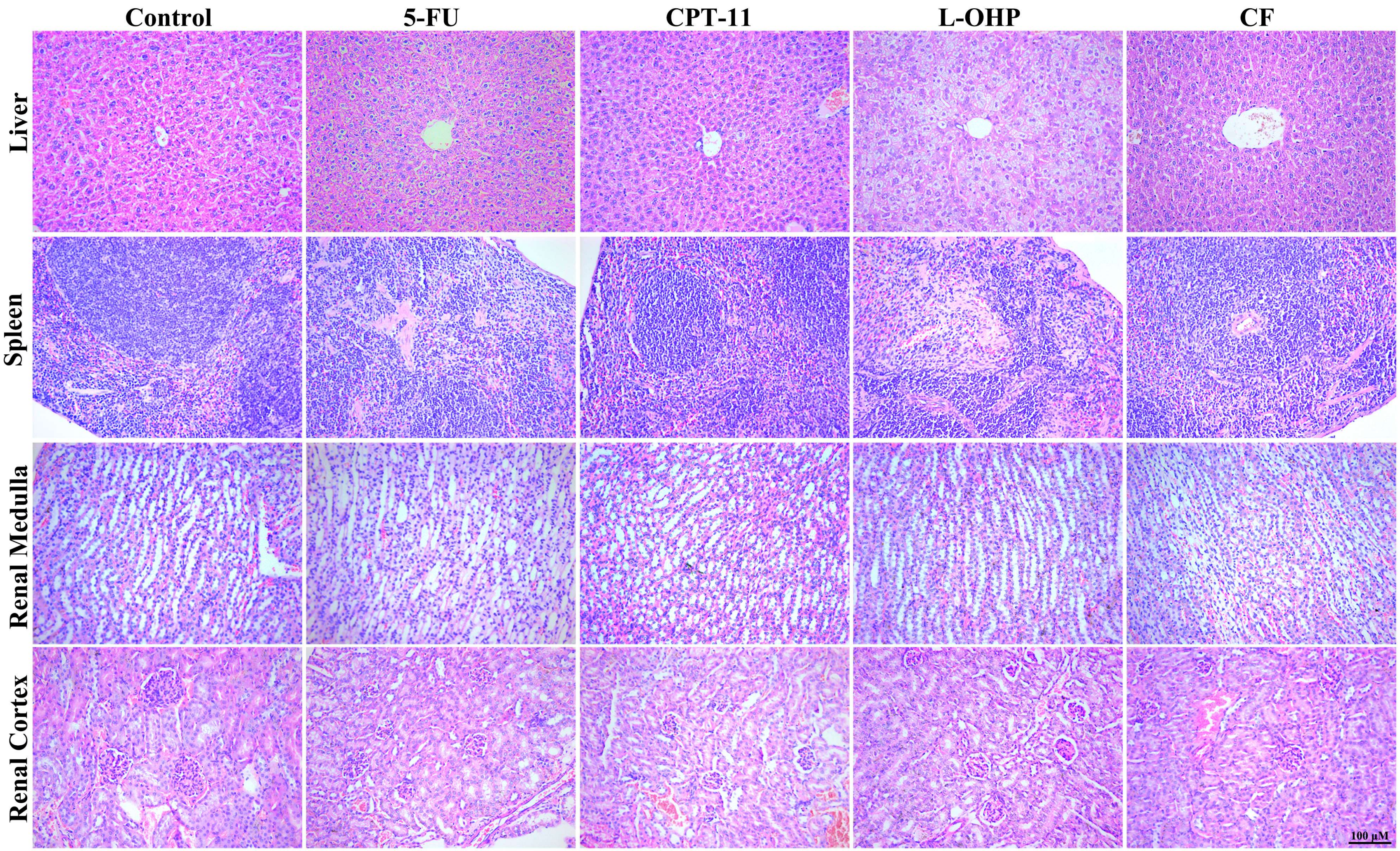

FIG S2 Photomicrographs of H&E-stained liver, spleen, renal medulla, and renal cortex sections. HE staining of representative histological sections of the liver, spleen, renal medulla and renal cortex from the groups (200× magnification).

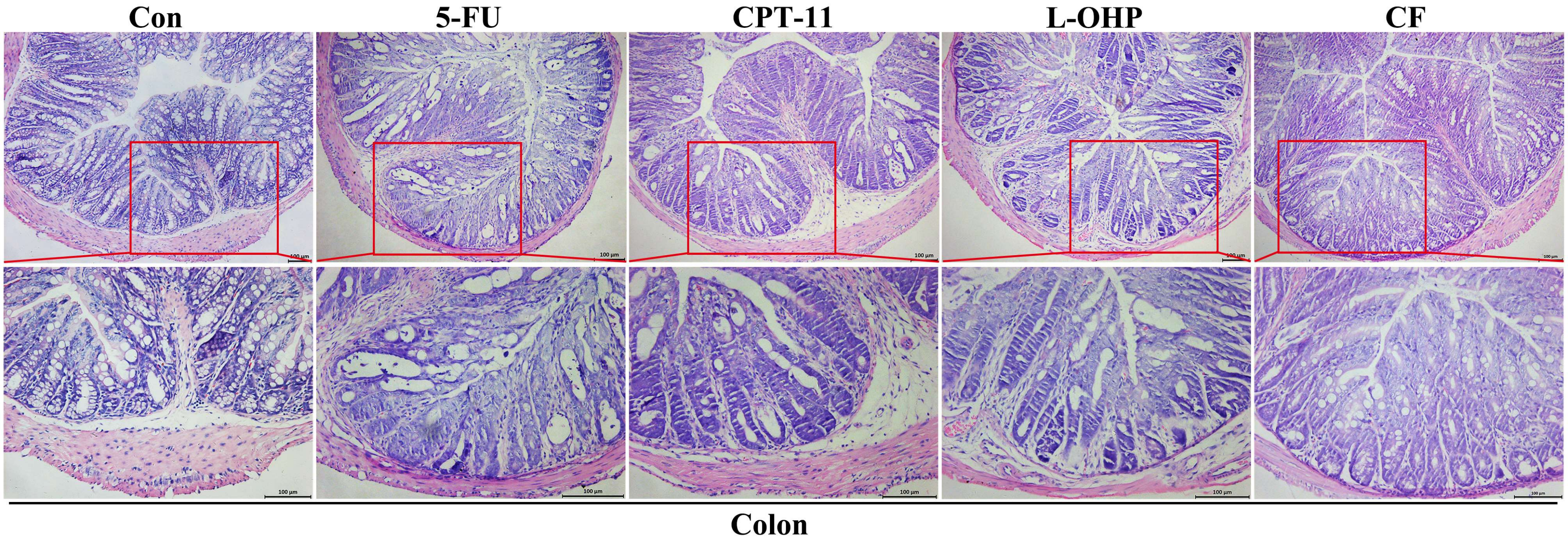

FIG S3 Photomicrographs of H&E-stained colon sections. HE staining of representative histological sections of the colon from the groups (200× magnification).

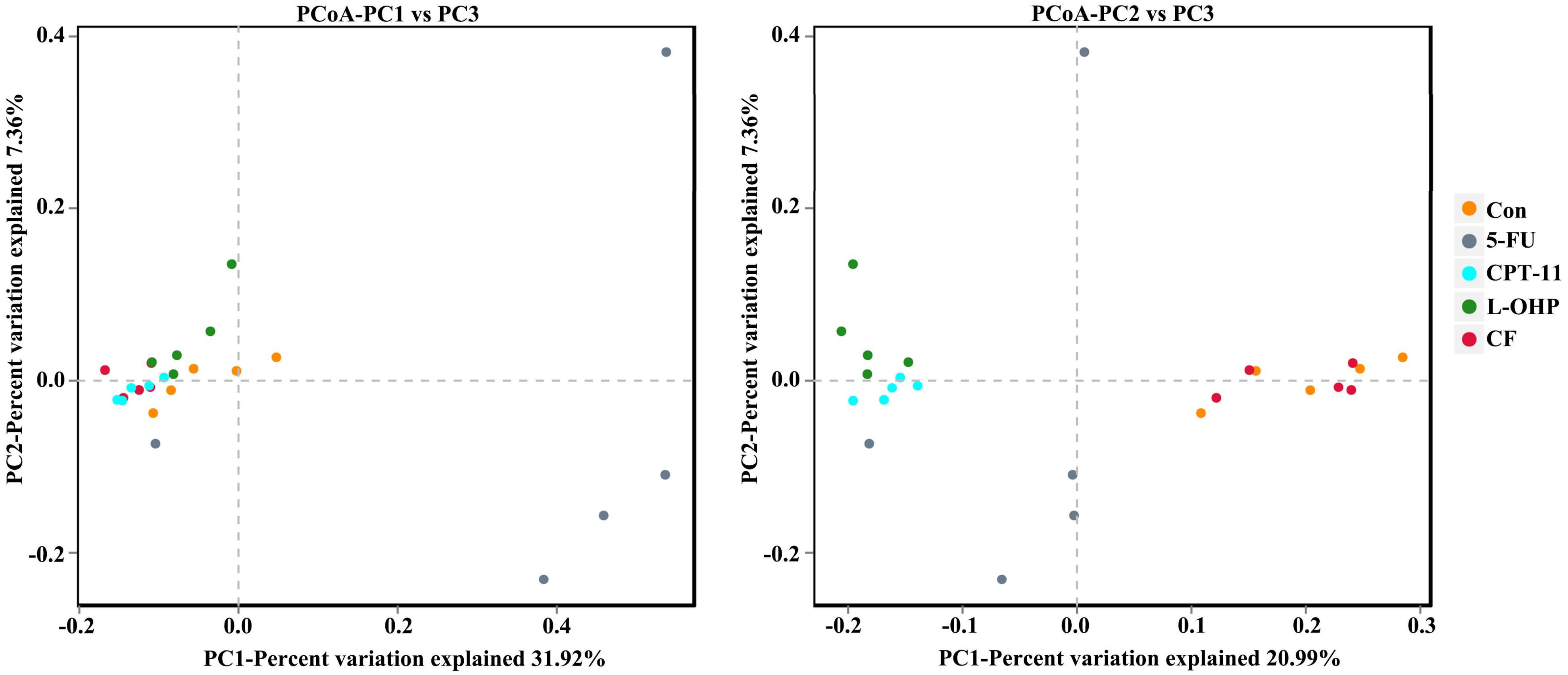

FIG S4 PCoA of different groups. Effects of different chemotherapeutic drugs on the gut microbiota, analysing by PCoA.

Supplementary Tables

Table S1

Score	Body Weight Loss	Diarrhea Index	Time Cost for detection of Fecal blood
0	≤1%	normal stool	≥2 min
1	1-5%	slightly wet and soft stool	1-2 min
2	6-10%	soft cylinder and easily spreadable	30 s-1 min
3	11-15%	wet and unformed stool	10 s-30 s
4	≥15%	non-cylindrical or runny	≤10 s or gross visibility of blood

Table S1 Criteria for scoring the body weight loss, diarrhea index, and FOB.

Table S2: Antibodies for western blot

Antibody	Cat No.	WB	Company
Zo-1	21773-1-AP	1/2000	Wuhan Sanying bio-technology co., Ltd., China
Occludin	27260-1-AP	1/2000	Wuhan Sanying bio-technology co., Ltd., China
NOD1	DF6378	1/1000	Cell Signaling Technology , USA
NOD2	DF12125	1/1000	Cell Signaling Technology , USA
RIP2	15366-1-AP	1/1000	Wuhan Sanying bio-technology co., Ltd. , China
p-AKT	mAb #4060	1/1000	Cell Signaling Technology, USA
AKT	mAb #4691	1/1000	Cell Signaling Technology, USA
p-p65	mAb #3033	1/1000	Cell Signaling Technology, USA
p65	mAb #8242	1/1000	Cell Signaling Technology, USA
GAPDH	60004-1-Ig	1/5000	Wuhan Sanying bio-technology co., Ltd., China
Mouse IgG [HþL]	SA00001-1	1/10000	Wuhan Sanying bio-technology co., Ltd., China
Rabbit IgG [HþL]	SA00001-2	1/10000	Wuhan Sanying bio-technology co., Ltd., China
